# Correction: Harpagophytum procumbens for osteoarthritis and low back pain: A systematic review

**DOI:** 10.1186/1472-6882-5-7

**Published:** 2005-03-21

**Authors:** Joel J Gagnier, Sigrun Chrubasik, Eric Manheimer

**Affiliations:** 1 Department of Health Policy Management and Evaluation, University of Toronto, Faculty of Medicine, Toronto, Canada; 2 Canadian College of Naturopathic Medicine, Academics, Toronto, Canada; 3 Department of Forensic Medicine, University of Freiburg, Freiburg, Germany; 4 Herbal Medicines Research and Education Center, Faculty of Pharmacy, University of Sydney, Sydney, Australia; 5 Center for Integrative Medicine, University of Maryland, School of Medicine, USA

After publication of this article [1], it was learned that there was an inadvertent omission of the following statement acknowledging the National Center for Complementary and Alternative Medicine funding, which supported Eric Manheimer's work on this article:

Eric Manheimer was funded by Grant Number R24 AT001293 from the National Center for Complementary and Alternative Medicine (NCCAM). The contents of this article are solely the responsibility of the authors and do not necessarily represent the official views of the NCCAM, or the National Institutes of Health.

We regret any inconvenience that the omission of this acknowledgment statement might have caused.

## Pre-publication history

The pre-publication history for this paper can be accessed here:


